# Regional variations in stiffness in live mouse brain tissue determined by depth-controlled indentation mapping

**DOI:** 10.1038/s41598-018-31035-y

**Published:** 2018-08-21

**Authors:** Nelda Antonovaite, Steven V. Beekmans, Elly M. Hol, Wytse J. Wadman, Davide Iannuzzi

**Affiliations:** 1 Department of Physics and Astronomy and LaserLab Amsterdam, Vrije Universiteit Amsterdam, De Boelelaan 1085, 1081 HV Amsterdam, The Netherlands; 2 Department of Translational Neuroscience, Brain Center Rudolf Magnus, University Medical Center Utrecht, 3584 CG Utrecht, The Netherlands; 3 Netherlands Institute for Neuroscience, An Institute of the Royal Netherlands Academy of Arts and Sciences, 1105 BA Amsterdam, The Netherlands; 4 Center for Neuroscience, Swammerdam Institute for Life Sciences, University of Amsterdam, 1098 XH Amsterdam, The Netherlands

## Abstract

The mechanical properties of brain tissue play a pivotal role in neurodevelopment and neurological disorders. Yet, at present, there is no consensus on how the different structural parts of the tissue contribute to its stiffness variations. Here, we have gathered depth-controlled indentation viscoelasticity maps of the hippocampus of acute horizontal live mouse brain slices. Our results confirm the highly viscoelestic nature of brain tissue. We further show that the mechanical properties are non-uniform and at least related to differences in morphological composition. Interestingly, areas with higher nuclear density appear to be softer than areas with lower nuclear density.

## Introduction

Brain tissue consists of neuronal cell bodies, their processes (dendrites and axons, myelinated or not, which form either sparse branches and arborizations or dense fiber bundles), the interconnecting extracellular brain matrix (ECM), glial cells, blood vessels, and extracellular fluid. Each of these components as well as their joint organization may have a different influence on the local mechanical properties of the tissue, which, in turn, regulate a wide variety of very relevant mechanotransduction phenomena. For instance, mechanical signals are known to play a role in multiple vital processes of neural cells^1,2^, whereas neuronal growth, neurite extension, arborization patterns, neuronal traction forces, and the stiffness of individual neurons and glial cells were all found susceptible to the stiffness of the substrate^3–11^. Furthermore, an abnormal mechanical environment (emerging as a result of internal or external forces, changes in the composition of the ECM, or changes in osmotic conditions) can disrupt normal brain function and neurodevelopment, and can alter progression of neurological disorders^12–14^. It is therefore commonly agreed that a deep knowledge of the correlation between brain composition and mechanical properties of the tissue would enable neuroscientists to shed light on how mechanotransduction phenomena contribute to the functioning of the brain. Furthermore, a quantitative assessment of the viscoelastic characteristics of the different regions of the brain could pave the way for the improvement of computational brain injury models^15^, the engineering of brain phantoms for surgical practise^16,17^, the design of mechanically matched brain implants^18^, and the production of soft substrates that could mimic different mechanical environments for investigations of stiffness-dependent neural stem cell differentiation^19–21^ and neuronal and glial cell morphology^22^. Yet, at present, the relation between mechanical properties and cytoarchitecture is still largely unknown.

Previous studies on brain mechanics have been mainly limited to the comparison between white and gray matter, with results that are either inconsistent or lack quantitative structure analysis^23–31^. Only recently, one study has shown that the mechanical properties correlate with myelin content in bovine brain^24^, while stiffness was found to scale with the cell nucleus area in spinal cord of mouse and retinal ruminant tissue^30,32^. Even though differences in mechanical properties of large hippocampal regions such as cornu ammonis (CA) fields and the dentate gyrus (DG) have been reported previously^33–37^, this set of data is not sufficient to completely determine the correlation between the morphological structure of the brain and its mechanical properties.

From a purely technical perspective, this literature gap is not surprising. Brain tissue is a highly viscoelastic, non-linear, anisotropic material^27,38–42^, and, because of its cellular heterogeneity, low stiffness, and rapid degradation, it is one of the most difficult (bio) materials to mechanically test. Macroscopic tests (such as shear rheology, compression testing, and tension testing) can only measure the mechanical properties of large samples, and, for this reason, cannot provide information on the local features of the tissue. Atomic force microscope (AFM) indentation, on the contrary, makes use of a small radius tip to locally probe the mechanical response of a material to a compressive stress, and, therefore, seems to be more suitable to assess how the mechanical properties of the different regions of the brain may be influenced by the underlying morphological composition^43^. Unfortunately, the results reported in the literature do not always agree with each other, as witnessed by the wide range of stiffness values reported^39,42,44^.

Tantalized by this challenge, we have explored whether a recently introduced mechanical testing technique, known as ferrule-top dynamic indentation^45,46^, could provide a better insight on the correlation between the composition of brain tissue and its viscoelastic properties. The technique used in our experiment is quite similar to AFM indentation. However, in our approach, the position of the cantilever is monitored by means of optical fiber interferometry rather than the optical triangulation technique used in AFM. As already showed in several papers^45–48^, this method is suited for the implementation of highly stable electromechanical feedback loops, which, in turn, guarantee better measurement protocols, including dynamic mechanical analysis at controlled indentation depth.

In this paper, we demonstrate that, using our deep, depth-controlled indentation method, one can obtain high spatial resolution viscoelasticity map of a mouse hippocampus slice. The map clearly emphasizes how the different structural regions give rise to different mechanical properties. Our results further show that brain tissue appears stiffer as the indentation depth or frequency increases–a result that confirms the non-linear viscoelastic nature of the brain tissue. Finally, calculating the mean measured stiffness of eleven anatomical subregions, and comparing it with the estimated nuclear densities, we can infer that densely packed cell layers may actually have lower stiffness than more disperse ones–a result that seems to contradict the commonly accepted assumption that brain tissue stiffness is dominated by cell bodies^3,30^.

## Results

### Mechanical heterogeneity of hippocampus agrees with anatomical region boundaries

**Figure 1:**
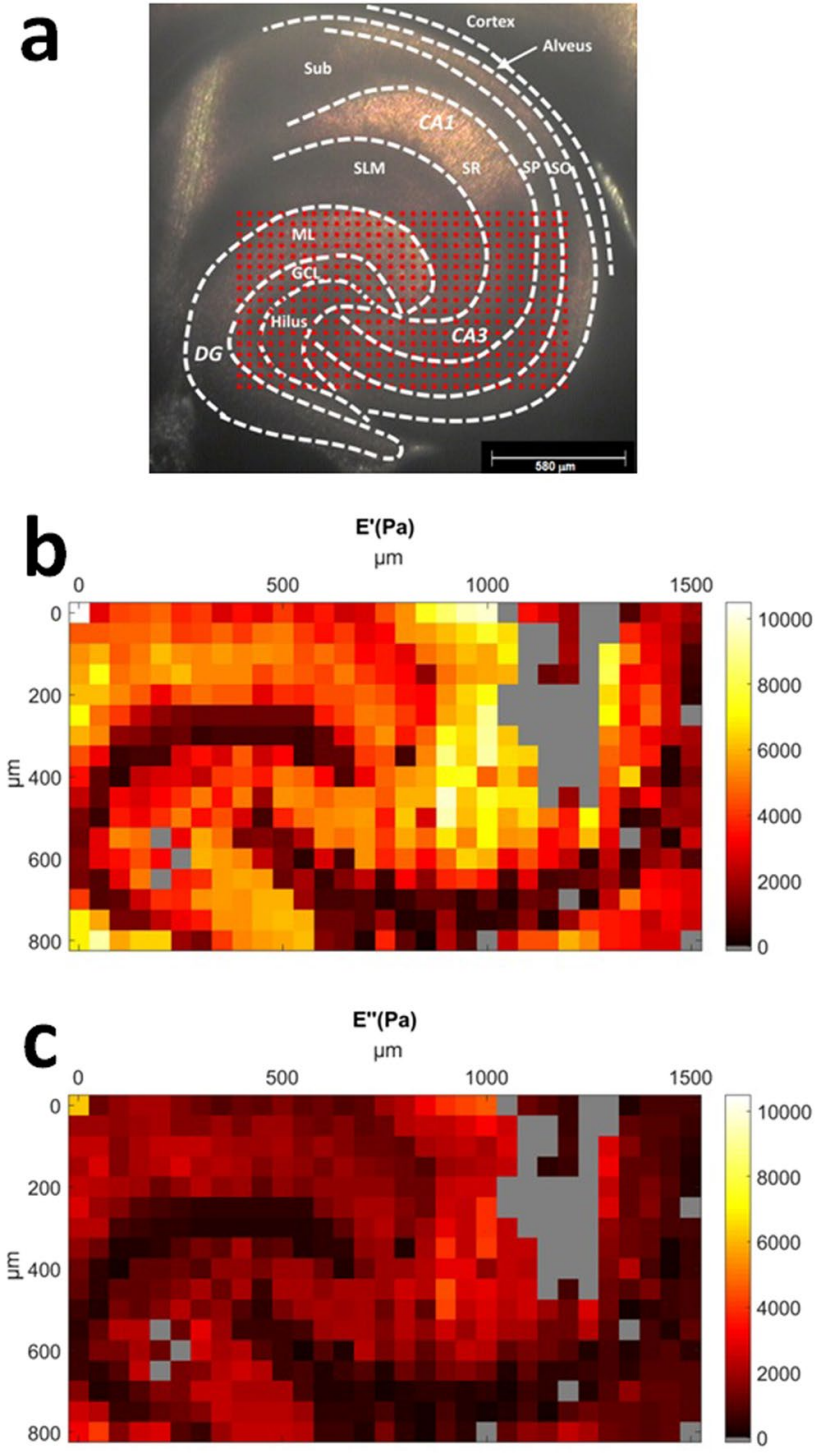
(**a**) Microscope image of one of the slices used in this experiment, along with a typical 50 × 50 μm indentation grid (red dots). Dashed lines indicate boundaries of morphologically distinct anatomical regions. Abbreviations: Sub - subiculum, SLM - stratum lacunosum moleculare, SR - stratum radiatum, SP - stratum pyramidale, SO - stratum oriens, ML - molecular layer, GCL - granule cell layer; large regions: DG - dentate gyrus, CA - cornus ammonis. (**b**,**c**) Color-coded map of storage *E*′ (**b**) and loss modulus *E*″ (**c**) in Pa over the DG and CA3 field of the hippocampal formation obtained with oscillatory ramp depth-controlled indentation strokes (see Methods) with 0.2 μm oscillation amplitude, 5.62 Hz oscillation frequency, and 9% strain. Gray color indicates failed measurements.

Viscoelasticity maps of live brain sections were obtained by means of ferrule-top depth-controlled indentation^45,46^. The image of one of the samples used in the experiment, along with a typical 50 × 50 μm grid of indentation locations, can be seen in Fig. 1a. We refer the reader to the method section for further details on instrumentation and protocol.

Figure 1b,c show the viscoelasticity maps (*E*′ = storage modulus; *E*″ = loss modulus) over the DG and the proximal portion of CA3 field of the hippocampus obtained from a horizontal mouse (9 months old) brain slice around 3 to 4 mm in the dorsal-ventral position. The representative maps were obtained with depth-controlled oscillatory ramp indentation strokes (see Methods) at an estimated strain of 9%. Similar maps, albeit focused on smaller areas, were obtained in 8 other slices from (7 slices from 6 months old mice and 1 slice from 9 months old mouse) out of 11 tested. As for the remaining 2 (both obtained from 9 months old mice), the data looked rather scrambled and not reproducible, probably because the sample was not perfectly adhering on the substrate.

In Fig. 1, one can clearly identify multiple areas with distinctive mechanical features. The shape of these regions agrees well with the morphological heterogeneity of anatomical subregions (see Fig. 1a), including the U-shaped structure of the molecular layer (ML) and of the granule cell layer (GCL) enclosing the hilus, and the laminar organization of strata (layers): oriens (SO), pyramidale (SP), radiatum (SR) and lacunosum-moleculare (SLM).

### Brain tissue is non-linear viscoelastic

**Figure 2:**
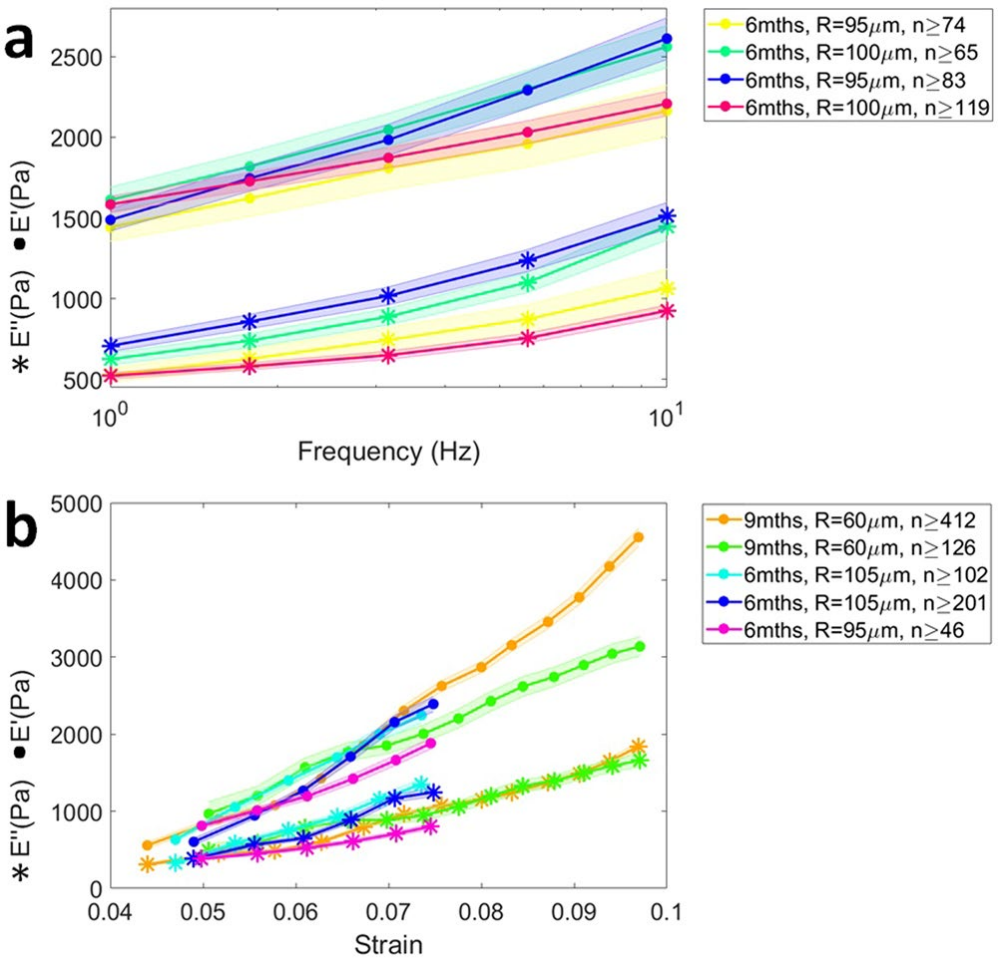
Non-linear viscoelastic properties of hippocampus obtained with dynamic indentation-controlled testing: frequency sweep (**a**) and oscillatory ramp (**b**). (**a**) Storage and loss moduli *E*′ and *E*″ increase over a frequency range of 1–10 Hz (data averaged over hippocampus; measured at ~7% strain, 0.2 μm oscillation amplitude; note the logarithmic scale on the x-axis). (**b**) *E*′ and *E*″ increase over the strain range of 4–10% (measured at 5.62 Hz oscillation frequency, 0.2 μm oscillation amplitude). The age of the animals is specified in the legend. Shadowed zones indicate the standard error of the mean, whereas *R* indicates the radius of the indenting tip and *n* the number of measurement points.

To confirm that ferrule-top depth-controlled dynamic indentation is capable of capturing the non-linear viscoelastic nature of the brain tissue, we first pooled all the data obtained from the hippocampus and performed joint analysis (i.e., without subdivision into layers). The averaged storage and loss moduli over frequency (obtained with the frequency sweep method) and strain (obtained with the oscillatory ramp method) are shown in Fig. 2. The frequency sweep data reveal a stiffening of the tissue with increasing indentation frequency, whereas the depth profiles from the oscillatory ramp testing show a stiffening with strain.

### Regional viscoelastic properties are reproducible

**Figure 3:**
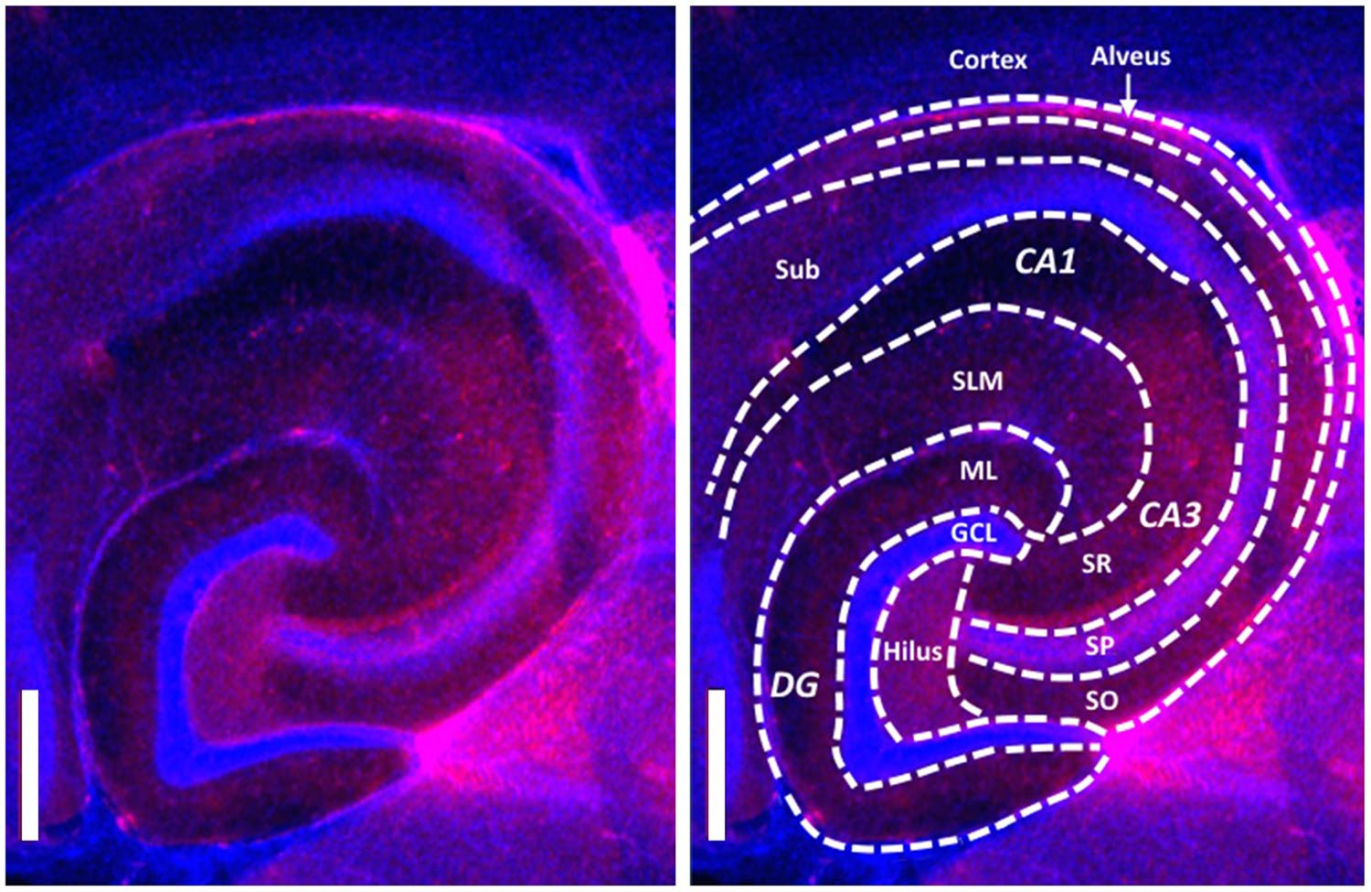
Fluorescent microscopy image of the hippocampus, visualizing cell nuclei (blue) and neurofilaments (red). Dashed lines indicate boundaries of morphologically distinct anatomical regions. Scale bar = 200 μm.

To test inter-animal variability of the mechanical properties measured in different brain areas, we developed a protocol to identify anatomical regions and indentation locations. The coordinates of the XYZ micromanipulator were calibrated, prior to the measurements, using the image of the tip of the probe in the camera of the inverted microscope. At the end of the indentation measurement, each brain section was formalin-fixed and stained for nuclei and neurofilaments, which indicates the neuronal axons (see Fig. 3, Methods). Differences in cell densities and organization of axons, visualized in fluorescent images, allowed us to draw the morphologically accepted boundaries between anatomical regions and overlay them with the image of the slice from the inverted microscope. Next, each indentation location was assigned to the corresponding anatomical region and the viscoelastic properties were averaged over these regions. The layered composition of the cortex areas varied between slices and were treated as a single region.

**Figure 4:**
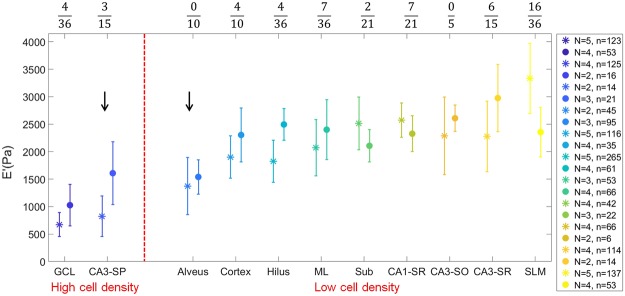
Comparison of the weighted means of the storage modulus of different brain regions obtained with an oscillatory ramp (stars) or a frequency sweep (dots) approach at 7.3% strain and 5.62 Hz frequency. Error bars are SE of the weighted mean. The legend indicates the number of slices *N* used to calculate the weighted mean and the total number of measurement points *n*. The fraction on top indicates the number of significantly different pairs over the total number of pairs analyzed with *post hoc* test. Arrows indicate regions with high content of fibers. Red dashed line separates two groups with low and high *A*_*rel*_.

Figure 4 shows the value of *E*′ at 7.3% strain and 5.62 Hz frequency, averaged over all the slices, for each of the different regions identified with the staining procedure, plotted from the softest to the stiffest in increasing stiffness order (as determined by the results obtained with the oscillatory ramp method). As expected from the viscoelasticity maps, the mechanical properties of the brain tissue appear highly heterogeneous. Both measuring methods (oscillatory ramp and frequency sweep) highlight the same trend in the mechanical properties of the different regions investigated, with the exception of the SLM region, where the frequency sweep method seems to indicate a decrease in stiffness that the oscillatory ramp does not detect.

To compare the local storage modulus measured in 9 slices from 8 animals, we performed one-way ANOVA followed by *post hoc* test for each region (Methods), where we used indentation measurements taken from various locations within the same region as testing samples. The results are indicated on top of Fig. 4 as a ratio between the number of significantly different pairs over the total number of pairs used for the comparison. Remarkably, only 17% of the tested pairs were significantly different, especially if one considers that 57% of these variations stems from the comparison of data obtained with different experimental methods (frequency sweep versus oscillatory ramp). This result confirms that, in our experiments, there is a good inter-animal reproducibility of the results.

### Higher relative area of nuclei yields lower stiffness

Our results strongly suggest a relation between stiffness and underlying morphological structure. As an attempt to establish a more objective analysis of the results obtained, we chose to focus on the percentage of area covered by cell nuclei as a mean to distinguish the different morphological regions. Three slices fluorescently labeled for nuclei were used to obtain approximate relative nuclei area *A*_*rel*_ in each measured region (see Methods). Based on the estimation, regions were divided into two groups: low (2.4–28.2%) and high (74.5–92.3%) *A*_*rel*_ (Fig. 4).

The low density regions appear to be stiffer than high density ones. The only exception seems to be the alveus, containing mostly fibers. One may speculate that high density of fibers may decrease the stiffness of the tissue in low *A*_*rel*_ regions. The high cell density region SP-CA3 also contains a lot of axons and has soft mechanical properties, which agrees with the idea that regions with high density of axons are soft. However, it is important to stress that, nuclear density is mainly intended as a way to substantiate known morphological differences between the investigated regions and it is not claimed to be the single causal parameter. Nevertheless, it seems clear that high nuclear density regions are softer than low density ones. Unfortunately, with data available, we cannot, as yet, draw robust conclusions on how this difference relates to more detailed morphological properties.

**Figure 5:**
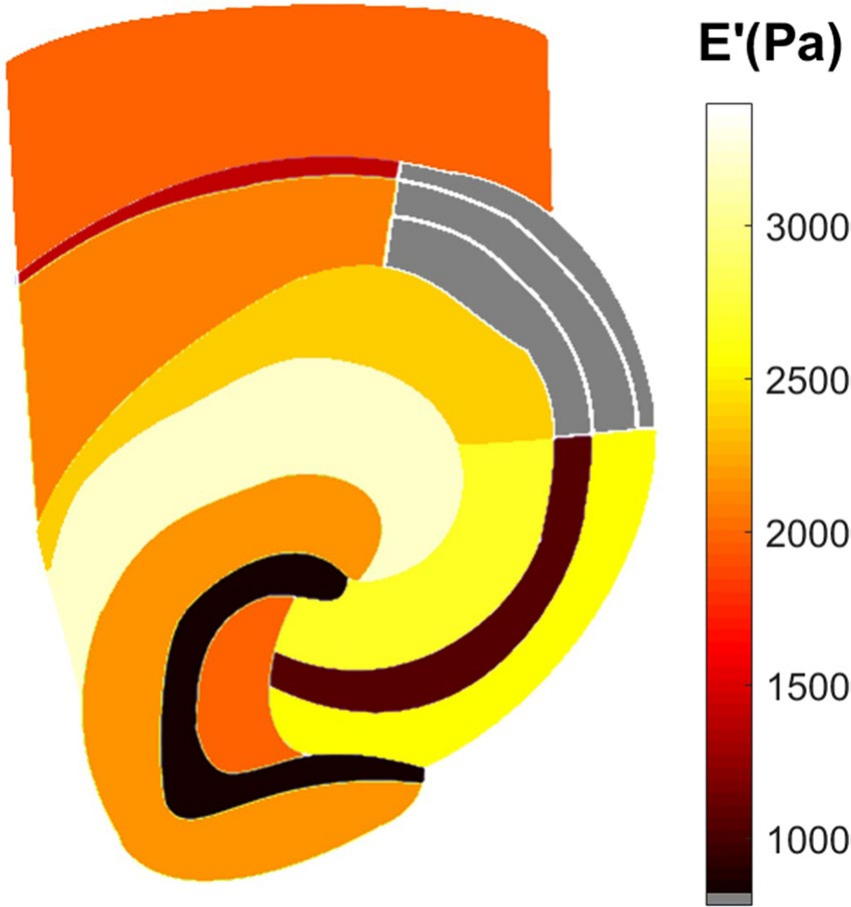
Reconstructed storage modulus map of brain tissue regions based on a weighted mean of 9 slices. Gray color indicates regions that were not measured.

For future reference, in Fig. 5, we provide a map of the storage modulus of the different brain tissue regions as reconstructed with the weighted means of 9 slices.

## Discussion

In this study, we have used a ferrule-top indentation approach to gather viscoelastic maps of mouse brain tissue *ex vivo*. The size of the indentation sphere and the depth of indentation were selected to ensure that the measurements could provide the tissue mechanical features of the subregional area of the tested sample. High spatial resolution (50 μm) of indentation mapping allowed us to find a clear correlation between indented regions and viscoelastic properties. The same relationship was reproduced on multiple slices by means of different testing method (frequency sweep and oscillatory ramp).

Our measurements show that both storage and loss moduli increase with strain. We can thus confirm that brain tissue is a non-linear material, as already reported in other studies^30,34,36^. Furthermore, performing the first localized frequency-domain indentation measurements on brain slices ever reported in the literature, we could observe that, in the 1–10 Hz range, both storage and loss moduli increase with indentation frequency–a viscoelastic behavior that was previously observed as a stress relaxation, creep response and change in strain-rate sensitivity in other indentation experiments^23,25,34,35,49^. In quantitative terms, it is interesting to note that, converting the averaged values of *E*′ obtained in our measurements (Fig. 2a) to shear moduli *G*′ by dividing *E*′ by 2(1 + *ν*) (where *ν* = 0.5 is the Poisson’s ratio of compressibility), one obtains values for *G*′ of 0.5–0.8 ± 0.1 kPa, which is in good agreement with macroscopic (i.e., not localized) frequency sweep measurements reported in the literature^28,31,50–56^. A direct comparison with other local measurements is unfortunately not possible, because the latter have been either performed on different kind of samples (different age, species, slicing direction) or under very diverse indentation stroke protocols, which, because of the highly viscoelastic behavior of the material, give rise to very different results^23–26,29,33–36,57^.

Our viscoelasticity maps on hippocampal subregions and the cortex further reveal that regions with higher cell body density corresponds to a softer tissue and vice versa. This is consistent with the study on single cortical neurons where soma has been found to be significantly softer than neurites^58^. However, our findings are in contrast with previous studies, which found a positive correlation between stiffness and cell nucleus area on the spinal cord of the adult mouse, retina of the ruminant and embryonic brain of *Xenopus*^3,30,32^. Furthermore, granular cell layer in coronal hippocampal slices of the juvenile rats was also shown to be stiffer than hilus^21^. While different CNS tissue composition might be the reason for the discrepancy between our results and those reported in the literature, it is worth stressing that our indentation protocol significantly differs with respect to that used in previous studies. The AFM system used in previous studies relies on small beads (radius <20 μm) and on a stroke that moves the probe at a constant speed of 10 μm/s until a predefined maximum force is reached (with the maximum force being on the order of several nN). We estimate that the contact radius must then be smaller than 10 μm, with an indentation depth of less than 4 μm. It is thus legitimate to ask whether this kind of AFM measurements probe the tissue properties or, actually, only indent the first layer of cells that lie on the surface which are also damaged during the slicing procedure^59^. Furthermore, the piezo-control testing used in AFM measurements results in different strain rates and indentation depth for different values of stiffness of the tissue indented; under the same stroke protocol, a softer tissue will experience a higher strain rate and a larger indentation depth. In contrast, our deep, indentation-control testing protocol assures constant indentation depth and constant indentation speed. Furthermore, with indentation beads in the range of 60–105 μm, strain of ~7%, and indentation depth between 8–11 μm (which results in a contact radius between 20 and 40 μm), we are sure to measure at the scale of the tissue. Therefore, we suggest that the opposite relationship between stiffness and areas of cell nuclei observed in our experiment might be at least partially due to the difference in the scale probed and/or in the very same testing method.

Our data is not sufficient to causally explain why low nuclear density regions translate into stiffer tissue, mainly because there are many morphological factors in the lower nuclear density regions that need to be evaluated separately and can all have a specific contribution to stiffness. At first, one may think that the increase of stiffness with the decrease of nuclear density be due to the role of the perineural nets (PNNs). However, it has been showed that this component lacks fibrous proteins^60^, and, therefore, should not support mechanical loading. Another component that may play a role in the mechanical properties of the brain tissue is myelin, as it has been shown that brain stiffness increases with myelin content^24,36^. Yet, our results indicate that the bundle of myelinated fibers in the alveus and the tract of mossy fibers along the SP in the CA3 field are actually softer. One may thus speculate whether the mechanical properties of the brain tissue as observed in our experiment are rather due to the fact that regions with low nuclear density host a larger amount of sparsely distributed axons and dendrites under tension^61–67^, which may in turn give rise to a stiffer material. To confirm or reject this hypothesis, it is imperative to perform new measurements that could directly correlate stiffness with axon density and orientation.

## Methods

### Sample preparation

C57Bl/6 mice were purchased from Harlan (Zeist, The Netherlands) and sacrificed at an age of 6 or 9 months. All experiments were performed in accordance with protocols and guidelines approved by the Institutional Animal Care and Use Committee (UvA-DEC) operating under standards set by EU Directive 2010/63/EU. All efforts were made to minimize the suffering and number of animals. The mice were decapitated, the brain was removed from the skull and stored in ice-cold ACSF containing (in mM): 120 NaCl, 3.5 KCl, 5 MgSO_4_, 1.25 NaH_2_ PO_4_, 2.5 CaCl_2_, 25 NaHCO_3_, and 10 glucose oxygenated with 95% O_2_/5% CO_2_ (~310 mOsmol/kg and ~pH 7.4). Slices were cut in a horizontal plane with a thickness of approximately 300 μm using a VT1200S vibratome (Leica Biosystems, Nussloch, Germany). Afterwards, a single brain tissue slice was placed in a perfusion chamber maintained at ~20 °C and supplied with carbogen saturated ACSF solution at 1 ml/min flow rate (gear pump MCP-Z standard, Ismatec). Lower than physiological mouse temperature was chosen to prolong the viability of the slice. The glass bottom of the chamber was coated with 0.05% polyethylenimine for the adhesion with the sample, which was gently mounted from the top with a 2 mm spaced harp to ensure the stability. After acclimatization for 1 h, indentation measurements were performed within 8 h. This time interval was chosen as the best compromise between number of measurements and overall durations of the experiment.

### Imaging and immunofluorescence

An inverted microscope (Nikon TMD-Diaphot, Nikon Corporation, Japan) was used to image the slice during the measurements with a 2x magnification objective (Nikon Plan 2X, Nikon Corporation, Japan). Images were recorded with a CCD camera (WAT-202B, Watec). After the measurements, slices were fixed in 4% PFA overnight at 4 °C. The sections were washed 3 × 10 min in PBS solution (0.01 M, pH = 7.4) and subsequently blocked for 2 h with 10% normal donkey serum (NDS), 0.25% Triton in PBS solution. After, slices were incubated overnight at 4 °C with primary antibody against neurofilament 160 kDa (NN18 N5264, Sigma Aldrich, 1:1000 dilution), 3% NDS and 0.25% Triton in PBS solution. Consequently, slices were washed 3 × 10 min in PBS solution and incubated for 2 h in PBS solution with DNA stain (Hoechst 33342, Invitrogen, dilution 1:2000), the secondary antibody Cy3-conjugated donkey anti-mouse IgG (Jackson Immuno Research, 1:1400 dilution), 1% NDS and 0.025% Triton. Finally, sections were washed 3 × 10 min in PBS and mounted with a glass coverslip in Vectashield (Vector Laboratories). Fluorescent images were obtained with Leica DMRE fluorescence microscope (Leica Microsystems, Wetzlar, Germany) and overlaid with the previously obtained bright-field images to identify anatomical regions of measured locations. Immunofluorescently-labeled slices were too thick for objective calculation of cell density and, thus, approximate percentage of area covered by nuclei was estimated for each region:

1 $${A}_{rel}=\frac{{A}_{nuclei}}{{A}_{total}},$$

where *A*_*nuclei*_ is the area covered by nuclei in the region and *A*_*total*_ is the total area of that region (processed with image J).

### Dynamic indentation setup and measurement protocol

**Figure 6:**
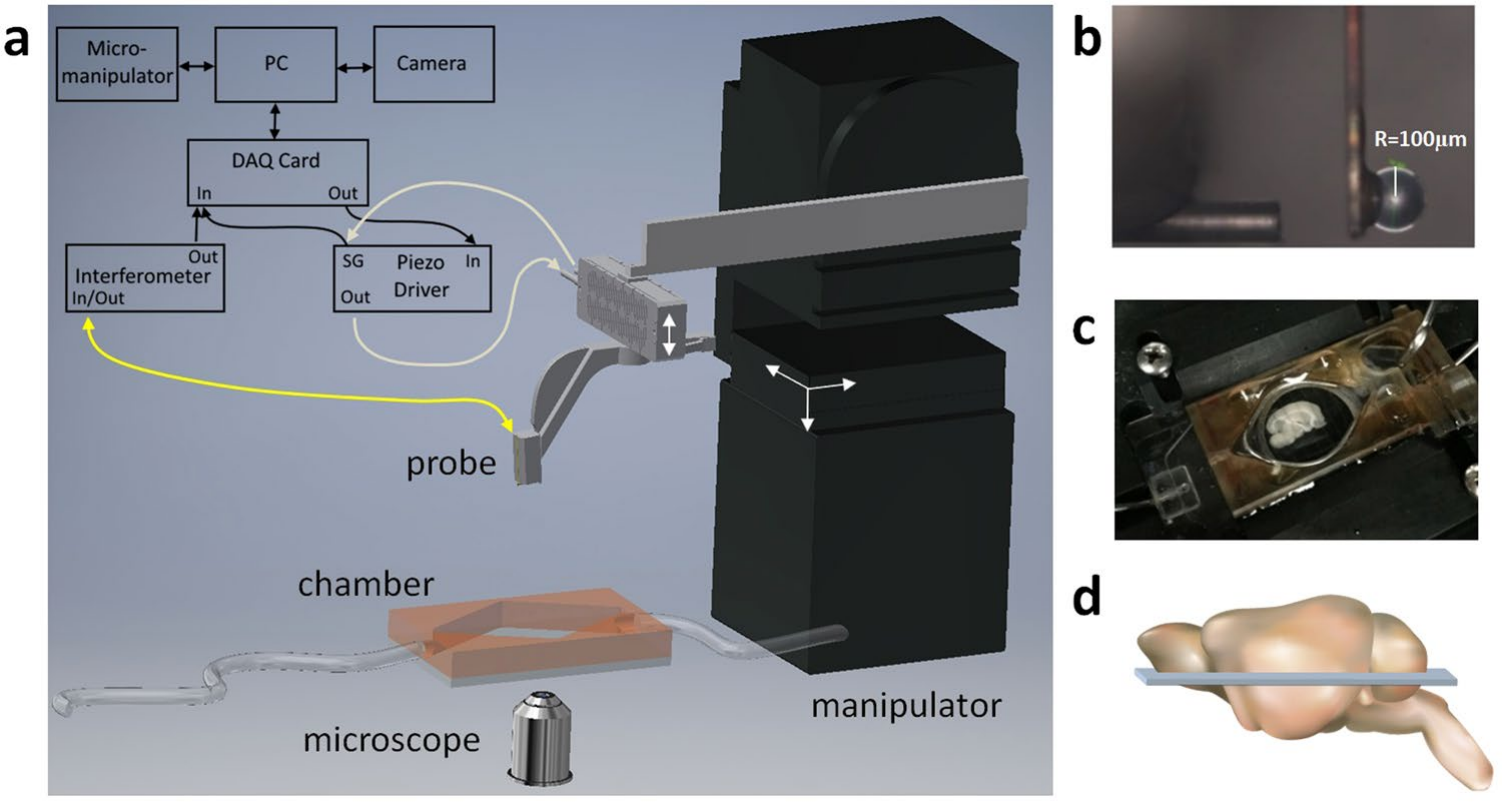
Schematic view of the dynamic indentation mapping setup. A ferrule-top probe (**a**) is equipped with an optical fiber for interferometric readout of the cantilever position and with a spherical tip to indent the sample (**b**). The probe is mounted on a piezoelectric transducer (**a**) for controlled movement during an indentation measurement. A brain slice is submerged in the perfusion chamber and fixed with the harp (**c**). The slice is imaged with an inverted microscope (**a**) for the determination of indentation locations. (**d**) The approximate position of the slice within the brain (the image is adapted from TogoTV^70^).

Horizontal mouse brain slices from 3 to 4 mm of dorsal-ventral positions of hippocampus (Fig. 6d) were submerged in a perfusion chamber assembled on the microscope, stabilized with a 2 mm spaced harp, and supplied with a constant flow of carbogenated artificial cerebrospinal fluid (ACSF). Measurements were carried out on 9 slices from eight mice of which 6 were 6 months old and 2–9 months old. The indentation lines were selected to cross the DG and the subiculum or the CA3 field of hippocampus (*n* ≥ 66 measurement points per slice). In addition, indentations on cortex were performed on 5 of the same slices adjacent to subiculum, in parallel lines between outer and inner layers (*n* ≥ 21 measurement points per slice).

A ferrule-top force transducer^45^, consisting of a micromachined cantilever spring with optical fiber readout, was mounted on a 3D printed holder screwed to a Z-piezoelectric actuator (PI p-603.5S2, Physik Instrumente). The single-mode fiber of the readout was coupled to an interferometer (OP1550, Optics11), where the interference signal was directly translated into cantilever deflection. Indentation depth control was implemented through a feedback loop, based on the error signal of cantilever deflection (Fig. 6, for more details see^46^). The piezoelectric actuator with the probe was mounted on a XYZ micromanipulator (PatchStar, Scientifica) for automatic mapping of mechanical properties. Indentation mapping was performed in parallel lines, with 59–476 points per slice. Distance between two adjacent locations were in the range of 50–160 μm, which assured that deformed areas do not overlap. A custom-written LabVIEW software (National Instruments) was used to process signals and to control the instrument through a data acquisition card (PCIe-6361, National Instruments).

**Figure 7:**
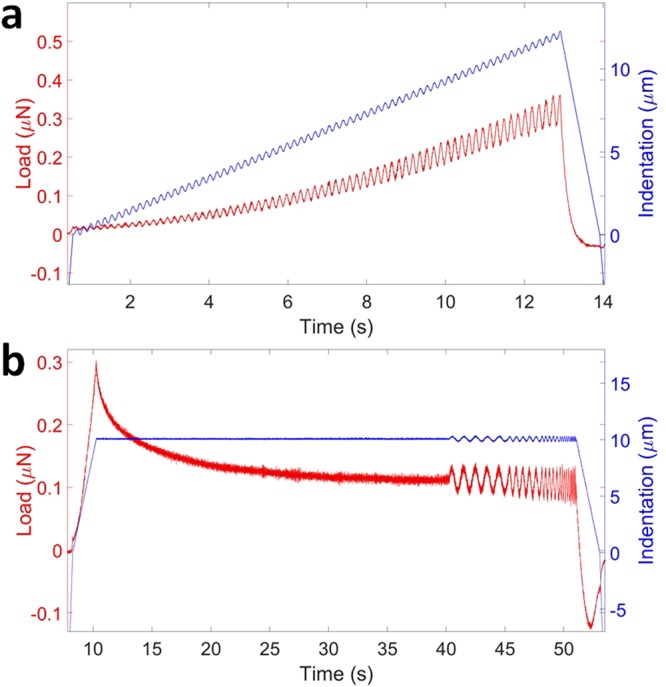
Depth-controlled indentation profiles. (**a**) Oscillatory ramp loading profile at 5.62 Hz oscillation frequency and (**b**) equilibrium frequency sweep profile between 1–10 Hz frequency range with both oscillation amplitudes of 0.2 μm.

Ferrule-top probes of 0.2–0.5 N/m stiffness and 60–105 μm bead radius were selected for these experiments and calibrated according to the method we recently introduced^68^. Two indentation-controlled profiles were selected for the characterization of depth and frequency dependent viscoelasticity: oscillatory ramp loading (OR) and equilibrium frequency sweep (FS). Figure 7 shows the typical curves of the controlled-indentation and load response. Depth-controlled oscillatory ramp indentations (Fig. 7a) had small 0.2 μm oscillations at 5.62 Hz frequency superimposed on top of a loading ramp at 0.01 strain rate estimated by $\dot{\varepsilon } \sim {\rm{\Delta }}\varepsilon /t$. Depth-controlled equilibrium frequency sweep measurements (Fig. 7b) consisted of the loading part up to 10 μm with 10 μm/s indentation speed, followed by 30 s stress relaxation period to reach mechanical equilibrium and series of small (*h*_0_ = 0.2 μm) sinusoidal oscillations at five distinct frequencies: 1, 1.78, 3.2, 5.62 and 10 Hz. The approach speed was set to 30 μm/s, the surface of the sample was determined, and an indentation-controlled feedback was triggered at approximate load of 15 nN, which resulted in the initial uncontrolled 1–3 μm indentation depth, which was later corrected in post processing procedures.

### Data analysis

Raw data was analyzed with custom-written MATLAB functions. The Hertz model was used to fit an initial loading data up to the cantilever threshold value to obtain the true surface position:

2 $$F=\frac{4}{3}\frac{E}{1-{\nu }^{2}}\frac{R}{\sqrt{{h}^{3}}},$$

where *F* is the load, *E* is an elastic modulus, *ν* is the Poisson’s ratio of compressibility (we assume that brain is incompressible *ν* = 0.5), *h* is the indentation depth. This allows us to correct the indentation depth *h* and to estimate the strain for measurements with probes of different radius: *ε* = 0.2 × *a*/*R*, where contact radius was estimated as $a=\sqrt{hR}$ varying between 22–39 μm. Strain of 7.3% was selected for comparative analysis in order to fulfill small strain approximation *ε* < 0.08^69^. While contact adhesion was observed as a pull-off force during retraction, it was not taken into account, as we assume that, under deep indentation conditions, the nominal area dominates over the actual one.

The sinusoidal oscillations were fit to cosine function, obtained amplitudes and phases were used to calculate the storage and loss moduli *E*′ and *E*″^46^, which is a measure of elasticity and viscosity, respectively:

3 $$\frac{E^{\prime} (\omega )}{1-{\nu }^{2}}=\frac{{F}_{0}}{{h}_{0}}cos\delta \frac{\sqrt{\pi }}{2}\frac{1}{\sqrt{A}},$$

4 $$\frac{E^{\prime\prime} (\omega )}{1-{\nu }^{2}}=\frac{{F}_{0}}{{h}_{0}}sin\delta \frac{\sqrt{\pi }}{2}\frac{1}{\sqrt{A}}$$

where *ω* is the frequency, *F*_0_ and *h*_0_ are the amplitudes of oscillatory load and indentation depth, respectively, *δ* is the phase-shift between the recorded indentation and load oscillations, *A* = *πa*^2^ is the contact area.

The contact area changes with the depth during oscillatory ramp, thus every 5 cycles were used for fitting and averaged indentation depth was used for the calculation of *E*′ and *E*″. Finally, all cosine fits with the *R*^2^ ≤ 0.7 and measurements which started in contact were rejected.

Normality of data distribution was tested with Shapiro-Wilk test (*n* ≥ 3). In case of normal distribution, statistical differences between multiple groups were investigated with one-way ANOVA followed by Bonferroni *post hoc* test to achieve 95% confidence level (*α* = 0.05). For non-normally distributed data, Kruskal-Wallis ANOVA test followed by Dunn’s *post hoc* test with *Š*id*á*k correction for *α*_1_ = 1 − (1 − *α*)^1/*k*^ was used to compare multiple groups. All statistical analyses were performed with Statistics and Machine Learning Toolbox (version 2017a, The Mathworks, Natick, MA, USA).

### Code availability

The computer code used to generate the results of this study is available on request from the corresponding author.

## Data Availability

All raw and processed data that support the findings of this study are available from the corresponding author upon request.

## References

[1] Moshayedi P (2014). The relationship between glial cell mechanosensitivity and foreign body reactions in the central nervous system. Biomaterials.

[2] Jagielska A (2012). Mechanical environment modulates biological properties of oligodendrocyte progenitor cells. Stem Cells and Development.

[3] Koser DE (2016). Mechanosensing is critical for axon growth in the developing brain. Nature Neuroscience.

[4] Moshayedi P (2010). Mechanosensitivity of astrocytes on optimized polyacrylamide gels analyzed by quantitative morphometry. Journal of Physics. Condensed Matter: An Institute of Physics Journal.

[5] Cullen DK, Lessing MC, LaPlaca MC (2007). Collagen-dependent neurite outgrowth and response to dynamic deformation in three-dimensional neuronal cultures. Annals of Biomedical Engineering.

[6] Georges PC, Miller WJ, Meaney DF, Sawyer ES, Janmey PA (2006). Matrices with compliance comparable to that of brain tissue select neuronal over glial growth in mixed cortical cultures. Biophysical Journal.

[7] Teixeira AI (2009). The promotion of neuronal maturation on soft substrates. Biomaterials.

[8] Sur S, Newcomb CJ, Webber MJ, Stupp SI (2013). Tuning supramolecular mechanics to guide neuron development. Biomaterials.

[9] Flanagan, L. A., Ju, Y.-E., Marg, B., Osterfield, M. & Janmey, P. A. Neurite branching on deformable substrates. *Neuroreport* **13**, 2411–2415, http://www.ncbi.nlm.nih.gov/pmc/articles/PMC2408859/ (2002). 10.1097/01.wnr.0000048003.96487.97 PMC2408859 12499839

[10] Bollmann, L. *et al*. Microglia mechanics: immune activation alters traction forces and durotaxis. *Frontiers in Cellular Neuroscience* **9**, http://www.ncbi.nlm.nih.gov/pmc/articles/PMC4585148/ (2015). 10.3389/fncel.2015.00363 PMC4585148 26441534

[11] Chen, L., Li, W., Maybeck, V., Offenhusser, A. & Krause, H.-J. Statistical study of biomechanics of living brain cells during growth and maturation on artificial substrates. *Biomaterials* **106**, 240–249, http://www.sciencedirect.com/science/article/pii/S0142961216304161 (2016). 10.1016/j.biomaterials.2016.08.029 27573132

[12] Kumar S, Weaver VM (2009). Mechanics, malignancy, and metastasis: the force journey of a tumor cell. Cancer Metastasis Reviews.

[13] Barros CS, Franco SJ, Müller U (2011). Extracellular matrix: functions in the nervous system. Cold Spring Harbor Perspectives in Biology.

[14] Vgh MJ (2014). Reducing hippocampal extracellular matrix reverses early memory deficits in a mouse model of Alzheimer’s disease. Acta Neuropathologica Communications.

[15] Mao H, Elkin BS, Genthikatti VV, Morrison B, Yang KH (2013). Why Is CA3 More Vulnerable Than CA1 in Experimental Models of Controlled Cortical Impact-Induced Brain Injury?. Journal of Neurotrauma.

[16] Forte, A. E., Galvan, S., Manieri, F., Rodriguez y Baena, F. & Dini, D. A composite hydrogel for brain tissue phantoms. Materials & Design **112**, 227–238, http://www.sciencedirect.com/science/article/pii/S0264127516312370 (2016).

[17] Chen SJ-S (2012). An anthropomorphic polyvinyl alcohol brain phantom based on Colin27 for use in multimodal imaging. Medical Physics.

[18] Spencer, K. C. *et al*. Characterization of Mechanically Matched Hydrogel Coatings to Improve the Biocompatibility of Neural Implants. *Scientific Reports* **7**, 1952, https://www.nature.com/articles/s41598-017-02107-2 (2017). 10.1038/s41598-017-02107-2 PMC5434064 28512291

[19] Saha, K. *et al*. Substrate Modulus Directs Neural Stem Cell Behavior. *Biophysical Journal* **95**, 4426–4438, http://www.ncbi.nlm.nih.gov/pmc/articles/PMC2567955/ (2008). 10.1529/biophysj.108.132217 PMC2567955 18658232

[20] Keung AJ, Dong M, Schaffer DV, Kumar S (2013). Pan-neuronal maturation but not neuronal subtype differentiation of adult neural stem cells is mechanosensitive. Scientific Reports.

[21] Luque, T., Kang, M. S., Schaffer, D. V. & Kumar, S. Microelastic mapping of the rat dentate gyrus. *Royal Society Open Science* **3**, http://www.ncbi.nlm.nih.gov/pmc/articles/PMC4852636/ (2016). 10.1098/rsos.150702 PMC4852636 27152213

[22] Puschmann TB (2013). Bioactive 3d cell culture system minimizes cellular stress and maintains the *in vivo*-like morphological complexity of astroglial cells. Glia.

[23] Budday S (2015). Mechanical properties of gray and white matter brain tissue by indentation. Journal of the Mechanical Behavior of Biomedical Materials.

[24] Weickenmeier, J. *et al*. Brain stiffness increases with myelin content. *Acta Biomaterialia* **42**, 265–272, http://www.sciencedirect.com/science/article/pii/S1742706116303750 (2016). 10.1016/j.actbio.2016.07.040 27475531

[25] van Dommelen, J. A. W., van der Sande, T. P. J., Hrapko, M. & Peters, G. W. M. Mechanical properties of brain tissue by indentation: Interregional variation. *Journal of the Mechanical Behavior of Biomedical Materials* **3**, 158–166, http://www.sciencedirect.com/science/article/pii/S1751616109000940 (2010). 10.1016/j.jmbbm.2009.09.001 20129415

[26] Kaster, T., Sack, I. & Samani, A. Measurement of the hyperelastic properties of *ex vivo* brain tissue slices. *Journal of Biomechanics* **44**, 1158–1163, http://www.sciencedirect.com/science/article/pii/S0021929011000492 (2011). 10.1016/j.jbiomech.2011.01.019 21329927

[27] Feng, Y., Okamoto, R. J., Namani, R., Genin, G. M. & Bayly, P. V. Measurements of mechanical anisotropy in brain tissue and implications for transversely isotropic material models of white matter. *Journal of the Mechanical Behavior of Biomedical Materials* **23**, 117–132, http://www.sciencedirect.com/science/article/pii/S175161611300129X (2013). 10.1016/j.jmbbm.2013.04.007 PMC3752297 23680651

[28] Forte AE, Gentleman SM, Dini D (2017). On the characterization of the heterogeneous mechanical response of human brain tissue. Biomechanics and Modeling in Mechanobiology.

[29] Christ, A. F. *et al*. Mechanical difference between white and gray matter in the rat cerebellum measured by scanning force microscopy. *Journal of Biomechanics* **43**, 2986–2992, http://www.sciencedirect.com/science/article/pii/S0021929010003660 (2010). 10.1016/j.jbiomech.2010.07.002 20656292

[30] Koser, D., Moeendarbary, E., Hanne, J., Kuerten, S. & Franze, K. CNS Cell Distribution and Axon Orientation Determine Local Spinal Cord Mechanical Properties. *Biophysical Journal* **108**, 2137–2147, http://www.sciencedirect.com/science/article/pii/S0006349515003008 (2015). 10.1016/j.bpj.2015.03.039 PMC4423070 25954872

[31] Samadi-Dooki, A., Voyiadjis, G. Z. & Stout, R. W. An Indirect Indentation Method for Evaluating the Linear Viscoelastic Properties of the Brain Tissue. *Journal of Biomechanical Engineering* **139** (2017). 10.1115/1.4036486 28418454

[32] Weber, I., Yun, S.-H., Scarcelli, G. & Franze, K. The role of cell body density in ruminant retina mechanics assessed by atomic force and Brillouin microscopy. *Physical Biology*, 10.1088/1478-3975/aa6d18 (2017). 10.1088/1478-3975/aa6d18 PMC5952364 28406094

[33] Finan, J. D., Pearson, E. M. & Morrison, B. Viscoelastic properties of the rat brain in the horizontal plane. *Proceedings of the International Research Council on the Biomechanics of Injury conference* **40**, 474–485, http://www.safetylit.org/citations/index.php?fuseaction=citations.viewdetails citationIds[]=citjournalarticle_387901_38 (2012).

[34] Elkin BS, Ilankova A, Morrison B (2011). Dynamic, regional mechanical properties of the porcine brain: indentation in the coronal plane. Journal of Biomechanical Engineering.

[35] Elkin BS, Morrison B (2013). Viscoelastic properties of the P17 and adult rat brain from indentation in the coronal plane. Journal of Biomechanical Engineering.

[36] Elkin BS, Ilankovan A, Morrison B (2010). Age-dependent regional mechanical properties of the rat hippocampus and cortex. Journal of Biomechanical Engineering.

[37] Finan JD, Elkin BS, Pearson EM, Kalbian IL, Morrison B (2012). Viscoelastic Properties of the Rat Brain in the Sagittal Plane: Effects of Anatomical Structure and Age. Annals of Biomedical Engineering.

[38] Franceschini, G., Bigoni, D., Regitnig, P. & Holzapfel, G. A. Brain tissue deforms similarly to filled elastomers and follows consolidation theory. *Journal of Mechanics Physics of Solids* **54**, 2592–2620, http://adsabs.harvard.edu/abs/2006JMPSo.54.2592F (2006).

[39] Hrapko M, van Dommelen JaW, Peters GWM, Wismans JSHM (2008). The influence of test conditions on characterization of the mechanical properties of brain tissue. Journal of Biomechanical Engineering.

[40] Budday S (2017). Mechanical characterization of human brain tissue. Acta Biomaterialia.

[41] Labus KM, Puttlitz CM (2016). An anisotropic hyperelastic constitutive model of brain white matter in biaxial tension and structural-mechanical relationships. Journal of the Mechanical Behavior of Biomedical Materials.

[42] Chatelin, S., Constantinesco, A. & Willinger, R. Fifty years of brain tissue mechanical testing: From *in vitro* to *in vivo* investigations. *Biorheology* **47**, 255–276, http://content.iospress.com/articles/biorheology/bir576 (2010). 10.3233/BIR-2010-0576 21403381

[43] Elkin BS, Azeloglu EU, Costa KD, Morrison B (2007). Mechanical heterogeneity of the rat hippocampus measured by atomic force microscope indentation. Journal of Neurotrauma.

[44] Cheng S, Clarke EC, Bilston LE (2008). Rheological properties of the tissues of the central nervous system: a review. Medical Engineering & Physics.

[45] Chavan D (2012). Ferrule-top nanoindenter: An optomechanical fiber sensor for nanoindentation. Review of Scientific Instruments.

[46] Hoorn, H. v., A. Kurniawan, N., H. Koenderink, G. & Iannuzzi, D. Local dynamic mechanical analysis for heterogeneous soft matter using ferrule-top indentation. *Soft Matter* **12**, 3066–3073 http://pubs.rsc.org/en/Content/ArticleLanding/2016/SM/C6SM00300A (2016). 10.1039/c6sm00300a PMC4819682 26908197

[47] Beekmans, S. V., Emanuel, K. S., Smit, T. H. & Iannuzzi, D. Minimally Invasive Micro-Indentation: mapping tissue mechanics at the tip of an 18g needle. *Scientific Reports* **7**, https://www.ncbi.nlm.nih.gov/pmc/articles/PMC5595846/ (2017). 10.1038/s41598-017-10526-4 PMC5595846 28900134

[48] Beekmans SV, Iannuzzi D (2016). Characterizing tissue stiffness at the tip of a rigid needle using an opto-mechanical force sensor. Biomedical Microdevices.

[49] Finan, J. D., Sundaresh, S. N., Elkin, B. S., McKhann, G. M. & Morrison, B. Regional mechanical properties of human brain tissue for computational models of traumatic brain injury. *Acta Biomaterialia* **55**, 333–339, http://www.sciencedirect.com/science/article/pii/S1742706117302076 (2017). 10.1016/j.actbio.2017.03.037 28351681

[50] Brands DW, Bovendeerd PH, Peters GW, Wismans JS (2000). The large shear strain dynamic behaviour of *in-vitro* porcine brain tissue and a silicone gel model material. Stapp Car Crash Journal.

[51] Hrapko, M., van Dommelen, J. a. W., Peters, G. W. M. & Wismans, J. S. H. M. The mechanical behaviour of brain tissue: Large strain response and constitutive modelling. *Biorheology* **43**, 623–636, http://content.iospress.com/articles/biorheology/bir436 (2006). 17047281

[52] Peters, G. W. M., Meulman, J. H. & Sauren, A. A. H. J. The applicability of the time/temperature superposition principle to brain tissue. *Biorheology* **34**, 127–138, http://www.sciencedirect.com/science/article/pii/S0006355X97000097 (1997). 10.1016/S0006-355X(97)00009-7 9373395

[53] Shen F (2006). Modified Bilston nonlinear viscoelastic model for finite element head injury studies. Journal of Biomechanical Engineering.

[54] Chatelin, S., Vappou, J., Roth, S., Raul, J. S. & Willinger, R. Towards child versus adult brain mechanical properties. *Journal of the Mechanical Behavior of Biomedical Materials* **6**, 166–173, http://www.sciencedirect.com/science/article/pii/S1751616111002475 (2012). 10.1016/j.jmbbm.2011.09.013 22301186

[55] Garo A, Hrapko M, van Dommelen JaW, Peters GWM (2007). Towards a reliable characterisation of the mechanical behaviour of brain tissue: The effects of post-mortem time and sample preparation. Biorheology.

[56] Vappou J (2007). Magnetic resonance elastography compared with rotational rheometry for *in vitro* brain tissue viscoelasticity measurement. Magnetic Resonance Materials in Physics, Biology and Medicine.

[57] Prange MT, Margulies SS (2002). Regional, Directional, and Age-Dependent Properties of the Brain Undergoing Large Deformation. Journal of Biomechanical Engineering.

[58] Grevesse T, Dabiri BE, Parker KK, Gabriele S (2015). Opposite rheological properties of neuronal microcompartments predict axonal vulnerability in brain injury. Scientific Reports.

[59] Huang, S. & Uusisaari, M. Y. Physiological temperature during brain slicing enhances the quality of acute slice preparations. *Frontiers in Cellular Neuroscience* **7**, https://www.ncbi.nlm.nih.gov/pmc/articles/PMC3632751/ (2013). 10.3389/fncel.2013.00048 PMC3632751 23630465

[60] Bonneh-Barkay D, Wiley CA (2009). Brain Extracellular Matrix in Neurodegeneration. Brain Pathology.

[61] Essen, D. C. V. A tension-based theory of morphogenesis and compact wiring in the central nervous system. *Nature* **385**, 313–318, https://www.nature.com/nature/journal/v385/n6614/abs/385313a0.html (1997). 10.1038/385313a0 9002514

[62] Haslach, H. W. *et al*. Solidextracellular fluid interaction and damage in the mechanical response of rat brain tissue under confined compression. *Journal of the Mechanical Behavior of Biomedical Materials* **29**, 138–150, http://www.sciencedirect.com/science/article/pii/S1751616113002981 (2014). 10.1016/j.jmbbm.2013.08.027 24084652

[63] Franze K (2013). The mechanical control of nervous system development. Development (Cambridge, England).

[64] Franze, K. & Guck, J. The biophysics of neuronal growth. *Reports on Progress in Physics* **73**, 094601, http://adsabs.harvard.edu/abs/2010RPPh…73i4601F (2010).

[65] Xu, G., Bayly, P. V. & Taber, L. A. Residual stress in the adult mouse brain. *Biomechanics and modeling in mechanobiology* **8**, 253–262, http://www.ncbi.nlm.nih.gov/pmc/articles/PMC4605564/ (2009). 10.1007/s10237-008-0131-4 PMC4605564 18651186

[66] Xu G (2010). Axons Pull on the Brain, But Tension Does Not Drive Cortical Folding. Journal of Biomechanical Engineering.

[67] Hilgetag CC, Barbas H (2006). Role of Mechanical Factors in the Morphology of the Primate Cerebral Cortex. PLOS Computational Biology.

[68] Beekmans, S. V. & Iannuzzi, D. A metrological approach for the calibration of force transducers with interferometric readout. *Surface Topography: Metrology and Properties* **3**, 025004, http://stacks.iop.org/2051-672X/3/i=2/a=025004 (2015).

[69] Lin, D. C., Shreiber, D. I., Dimitriadis, E. K. & Horkay, F. Spherical indentation of soft matter beyond the Hertzian regime: numerical and experimental validation of hyperelastic models. *Biomechanics and modeling in mechanobiology* **8**, 345–358, http://www.ncbi.nlm.nih.gov/pmc/articles/PMC3615644/ (2009). 10.1007/s10237-008-0139-9 PMC3615644 18979205

[70] Database center for life science, http://togotv.dbcls.jp/ja/togopic.2013.25.html (2016). 17867293

